# Exploring the Antitumor Efficacy of N-Heterocyclic Nitrilotriacetate Oxidovanadium(IV) Salts on Prostate and Breast Cancer Cells

**DOI:** 10.3390/molecules29122924

**Published:** 2024-06-19

**Authors:** Katarzyna Chmur, Aleksandra Tesmar, Magdalena Zdrowowicz, Damian Rosiak, Jarosław Chojnacki, Dariusz Wyrzykowski

**Affiliations:** 1 Faculty of Chemistry, University of Gdańsk, Wita Stwosza 63, 80-308 Gdańsk, Poland; katarzyna.chmur@ug.edu.pl (K.C.); aleksandra.tesmar@ug.edu.pl (A.T.); magdalena.zdrowowicz@ug.edu.pl (M.Z.); 2 Department of Inorganic Chemistry, Gdańsk University of Technology, Narutowicza 11/12, 80-233 Gdańsk, Poland; damianrosiak91@gmail.com (D.R.); jaroslaw.chojnacki@pg.edu.pl (J.C.)

**Keywords:** vanadium complexes, crystal structure, prostate cancer cells, breast cancer cells, antitumor activity

## Abstract

The crystal structures of two newly synthesized nitrilotriacetate oxidovanadium(IV) salts, namely [QH][VO(nta)(H_2_O)](H_2_O)_2_ (**I**) and [(acr)H][VO(nta)(H_2_O)](H_2_O)_2_ (**II**), were determined. Additionally, the cytotoxic effects of four N-heterocyclic nitrilotriacetate oxidovanadium(IV) salts—1,10-phenanthrolinium, [(phen)H][VO(nta)(H_2_O)](H_2_O)_0.5_ (**III**), 2,2′-bipyridinium [(bpy)H][VO(nta)(H_2_O)](H_2_O) (**IV**), and two newly synthesized compounds (**I**) and (**II**)—were evaluated against prostate cancer (PC3) and breast cancer (MCF-7) cells. All the compounds exhibited strong cytotoxic effects on cancer cells and normal cells (HaCaT human keratinocytes). The structure–activity relationship analysis revealed that the number and arrangement of conjugated aromatic rings in the counterion had an impact on the antitumor effect. The compound (III), the 1,10-phenanthrolinium analogue, exhibited the greatest activity, whereas the acridinium salt (II), with a different arrangement of three conjugated aromatic rings, showed the lowest toxicity. The increased concentrations of the compounds resulted in alterations to the cell cycle distribution with different effects in MCF-7 and PC3 cells. In MCF-7 cells, compounds **I** and **II** were observed to block the G_2_/M phase, while compounds **III** and **IV** were found to arrest the cell cycle in the G_0_/G_1_ phase. In PC3 cells, all compounds increased the rates of cells in the G_0_/G_1_ phase.

## 1. Introduction

**Figure 1:**
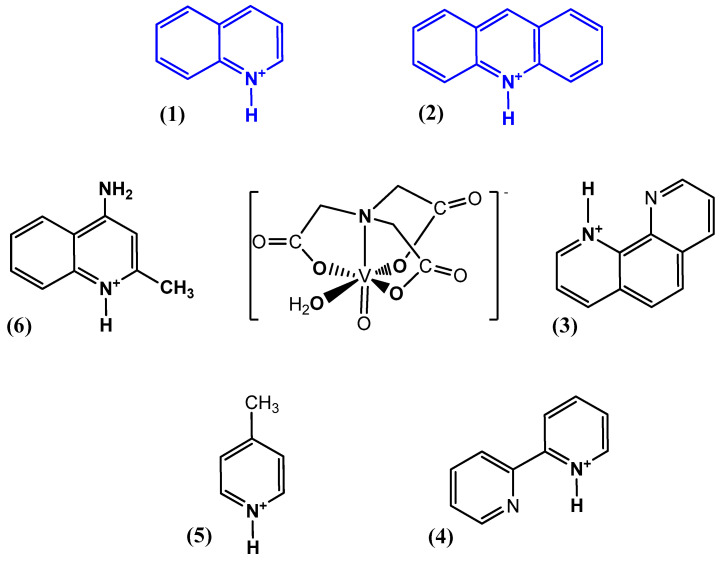
Examples of the counterions stabilizing discrete mononuclear [VO(nta)(H_2_O)]^−^ coordination units: (1) the quinolinium cation [QH]^+^, (2) the acridinium cation [(acr)H]^+^, (3) the 1,10-phenanthrolinium cation [(phen)H]^+^ [2], (4) the 2,2′-bipyridinium cation [(bpy)H]^+^ [3], (5) the 4-methylpyridinium cation [(4-Mepy)H]^+^ [4], and (6) the 4-amino-2-methylquinolinium cation [(4-NH_2_-2-MeQ)H]^+^ [5].

The nitrilotriacetate ligand (H_n_nta^(3−n)−^) has flexible coordination properties that allow for the construction of fairly stable oxidovanadium(IV) complexes, including heterometallic ones, with a range of interesting coordination entities. These types of V(IV) complex anions can exist as binuclear or mononuclear entities. However, unlike the previous units, which are stabilized in the solid state with inorganic cations [1], namely [(VO)_2_(μ_2_-O)(nta)_2_]^4−^ and [(VO)_2_(μ_2_-O)(nta)_2_M(H_2_O)_4_]^2−^, the presence in the crystal lattice of a distantly separated, monomeric anion, [VO(nta)(H_2_O)]^−^, is unique. Previous reports have shown that aqua-(nitrilotriacetato-N,O,O′,O″)-oxidovanadium(IV) anions are formed with protonated N-heterocyclic compounds (Figure 1). The observed phenomenon is subsequently confirmed in this paper with two other N-heterocyclic counterions, i.e., the quinolinium cation, [QH]^+^ (**I**), and acridinium cation, [(acr)H]^+^ (**II**).

Recently, several biological studies have shown that oxidovanadium(IV)-based complexes [6,7,8] have potential as anticancer agents. These complexes disrupt cellular metabolism and alter cellular organelles, such as lysosomes and mitochondria, which are the main targets for their antitumor effects. Additionally, vanadium can cause genotoxic effects on the nuclei of cells and DNA damage, which can also disturb cell proliferation [9]. Table 1 summarizes the biological activities of certain [VO(nta)(H_2_O)]^−^ salts with protonated N-heterocyclic cations.

However, the underlying anticancer mechanisms involving vanadium compounds remain to be elucidated [9,11,12]. Therefore, it is necessary to conduct systematic studies on the structure–activity relationships of this class of compounds in biological systems to gain a better understanding of their action.

In this paper, we present the crystal structures of two newly synthesized nitrilotriacetate oxidovandium(IV) salts with the quinolinium cation, [QH][VO(nta)(H_2_O)](H_2_O)_2_ (**I**), and the acridinium cation, [(acr)H)][VO(nta)(H_2_O)](H_2_O)_2_ (**II**). Additionally, to gain a better insight into the role of the counterions on the biological properties of the investigated salts, the cytotoxic effects of the compounds (**I**) and (**II**) as well as their 1,10-phenanthrolinium (**III**) and 2,2′-bipyridinium (**IV**) analogues on breast cancer cells (MCF-7), prostate cancer cells (PC3), and human keratinocytes (HaCaT lines) were assessed and compared. Breast cancer is the second leading cause of cancer death among women after lung cancer [13,14], whereas prostate cancer was the second most frequent and fifth major cause of cancer-associated death among men in 2020. These types of human cancers are among the 200 known varieties that pose major public health concerns [15]. Systematic studies on the structures and physicochemical and biological properties of the investigated complexes will allow for a more thorough search for vanadium-based complexes that are candidates for the formulation of pharmaceuticals with the desired biological properties.

## 2. Results and Discussion

### 2.1. Structural Description

**Figure 2:**
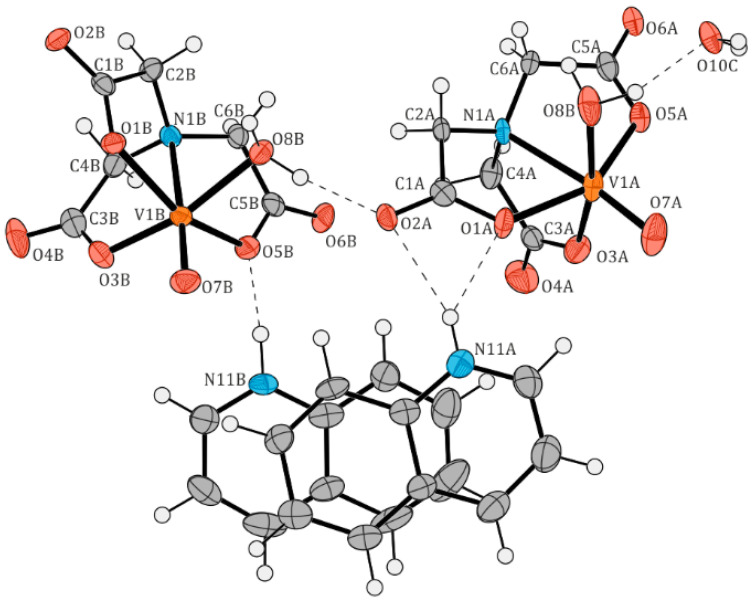
The molecular structure of [QH][VO(nta)(H_2_O)](H_2_O)_2_ (**I**). Displacement ellipsoids are drawn at the 50% probability level, hydrogen atoms are drawn as spheres of arbitrary radii. Atom colors: O—red, N—blue, C—grey, H—white.

**Figure 3:**
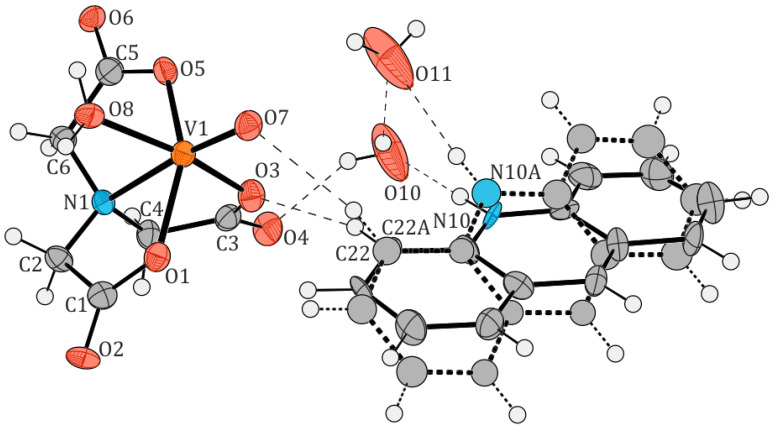
The molecular structure of [(acr)H][VO(nta)(H_2_O)](H_2_O)_2_ (**II**). Displacement ellipsoids are drawn at the 50% probability level, hydrogen atoms are drawn as spheres of arbitrary radii. The hydrogen bonds are indicated by dashed lines. Atoms belonging to the disordered part (thick dashed lines) were refined isotropically. Atom colors: O—red, N—blue, C—grey, H—white.

The crystal structures of two new oxidovanadium(IV) compounds, [QH][VO(nta)(H_2_O)](H_2_O)_2_ (**I**) and [(acr)H][VO(nta)(H_2_O)](H_2_O)_2_ (**II**), are shown in Figure 2 and Figure 3, respectively. Table 2 and Table 3 list selected interatomic bond distances and angles for both compounds. Tetragonal crystals of compound (**I**) consist of two aqua-(nitrilotriacetato-N,O,O′,O″)-oxidovanadium(IV) anions, [VO(nta)(H_2_O)]^−^, two quinolinium cations, [QH]^+^, and one water molecule. Three water molecules in the asymmetric unit of (**I**) were found to be highly disordered, with atoms in special positions, and could therefore not be satisfactorily modeled. They were removed from the electron density map using the OLEX solvent mask command. Compound (**II**) crystallizes in an orthorhombic system containing one [VO(nta)(H_2_O)]^−^ anion, one acridinium cation [(acr)H]^+^, modeled as disordered over two positions, and two water molecules in the asymmetric unit. In contrast, salts of [VO(nta)(H_2_O)]^−^ with the 1,10-phenanthrolinium cation [2], 2,2′-bipyridinium cation [3], and 4-methylpyridinium cation [4] crystallize in the monoclinic system. The crystal structure of the investigated complexes confirms the previous finding that protonated N-heterocyclic compounds acting as counter-ions prevent the assembling of these coordination units to dimers via an oxide bridge.

**Figure 4:**
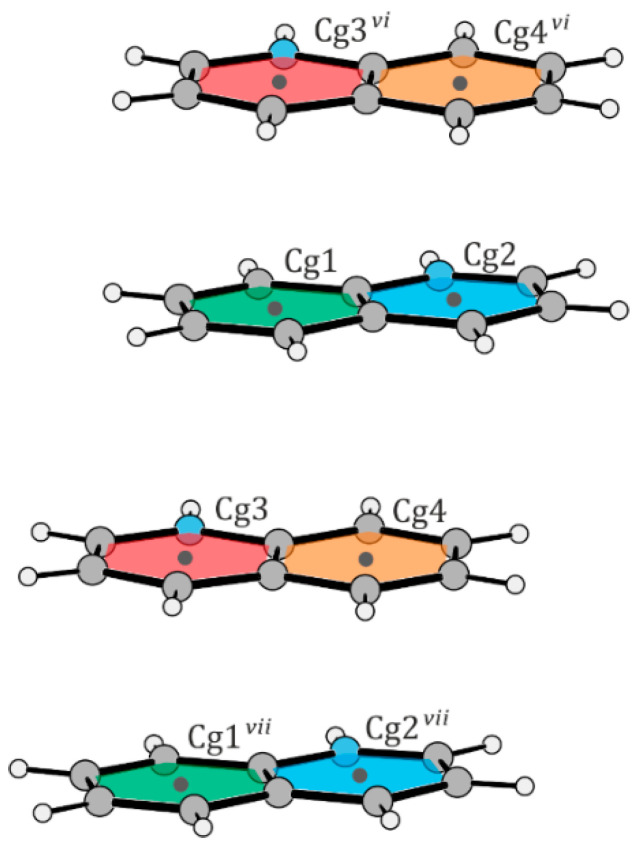
Schematic representation of layer interactions in structure (**I**). The centroids are marked with black dots. Cg1: (C14A-C19A); Cg2: (N11A, C11A-C14A, C19A); Cg3: (N11B, C11B-C14B, C19B); Cg4: (C14B-C19B). Symmetry transformations: (*vi*): *x*, *y*, − 1 + *z*; (*vii*): *x*, *y*, 1 + *z*.

**Figure 5:**
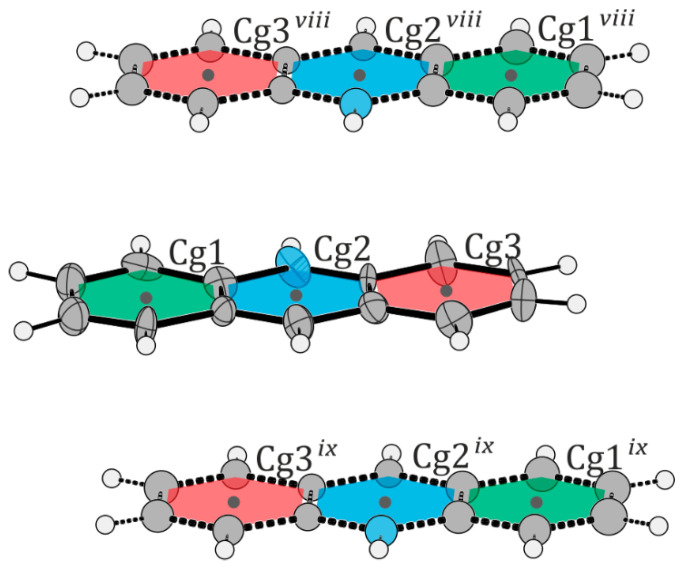
Schematic representation of layer interactions in structure (**II**). Atoms belonging to the disordered part (thick dashed lines) were refined isotropically. Cg1: (C11-C16); Cg2: (N10, C11, C16, C17, C18, C23); Cg3: (C18-C23). The centroids are marked with black dots. Symmetry transformations: *viii*: − ½+ *x*, ½ − *y*, 1 − *z*; *ix*: ½ + *x*, ½ − *y*, 1 − *z*.

The structural features of the [VO(nta)(H_2_O)]^−^ coordination units in compounds (**I**) and (**II**) are similar to those found in other compounds containing mononuclear nitrilotriacetate oxidovanadium(IV) moieties [2,3,4,5]: (i) the coordination sphere of the V^4+^ cation adopts a slightly distorted tetragonal bipyramidal geometry, (ii) the nitrogen atom of the nta^3−^ anion always occupies the *trans* position relative to the *oxido-* ligand, (iii) the V-N bond distance is longer than that found for dinuclear units containing a bridging oxygen atom (μ_2_-O), and (iv) the aqua ligand and the oxygen atoms of carboxylate groups occupy equatorial positions. Furthermore, the difference in wavenumber between the asymmetric and symmetric stretching vibrations of carboxylate groups in the IR spectra of the [VO(nta)(H_2_O)]^−^ salts is similar to that observed in the IR spectrum of the nitrilotriacetate sodium salt, Na_3_nta [2]. This confirms the involvement of carboxy groups in the coordination of the V^4+^ cation in a monodentate fashion and suggests the ionic nature of these interactions [16]. The O–H···O, N–H···O, C–H···N, and C–H···O intermolecular hydrogen bonds between the oxygen atoms of nitrilotriacetato- and aqua ligands and non-coordinated water molecules (Table 4 and Table 5) as well as the π···π stacking interactions of the neighboring organic cations play an important role in the stabilization of the crystal lattice of the investigated complexes. In structure (**I**), the two quinolinium cations located in the asymmetric unit form layer-type interactions with each other. Additionally, they stack with other quinolinium cations, extending along the crystallographic *c*-axis (Figure 4 and Table 6). In structure (**II**), the acridinium cations also participate in layer-type interactions, contributing to the disorder observed in the acridinium cation. These cations form stacks extending along the crystallographic *a*-axis. Both arrangements of the acridinium cation are stabilized by N–H···O hydrogen bonds, each interacting with a different water molecule (Figure 5 and Table 7). The water molecules, extracted from the electron density, form channels extending along the crystallographic *c*-axis.

### 2.2. Cytotoxic Activity of Vanadium Complexes

**Figure 6:**
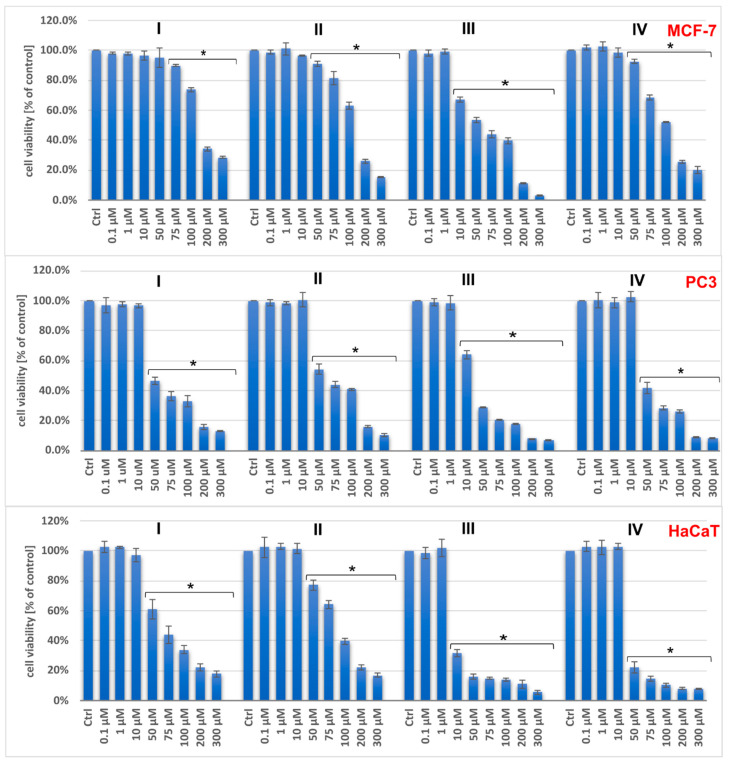
The viability of PC3, MCF-7, and HaCaT cells assessed by MTT assay after 48 h treatment with tested compounds (I–IV). Results are shown as the mean ± standard deviation (SD) of three independent experiments performed in triplicate. * Statistically significant difference is present between treated cultures and the control (untreated culture), *p* < 0.05.

**Figure 7:**
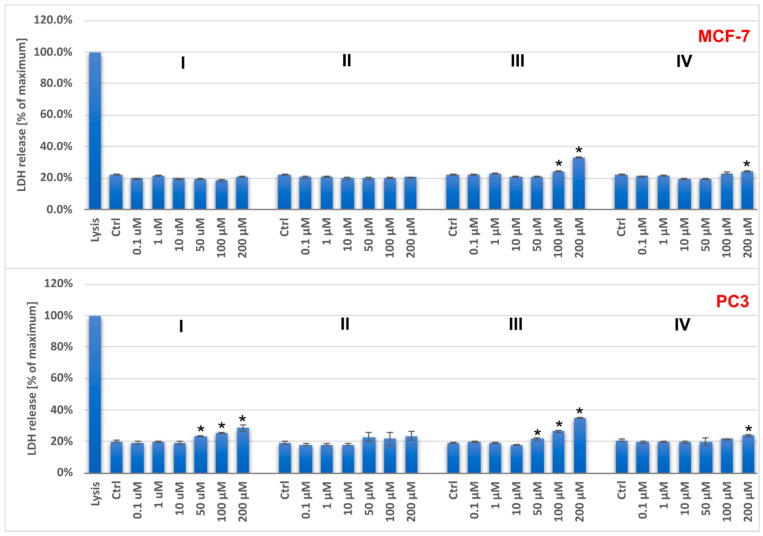
The LDH release for PC3 and MCF-7 cells after 48 h treatment with the tested compounds (I–IV). Results are shown as the mean ± standard deviation (SD) of three independent experiments performed in triplicate. * Statistically significant difference is present between treated cultures and the control (untreated culture), *p* < 0.05.

The cytotoxicity of the investigated salts on breast cancer cells (MCF-7), prostate cancer cells (PC3), and human keratinocytes (HaCaT lines) was tested at the mitochondrial level (the MTT assay) and the plasma membrane level (the LDH leakage). The results showed a dose-dependent anticancer effect for all compounds (Figure 6 and Figure 7).

The IC_50_ values indicate that the tested compounds have greater antitumor activity against PC3 cells than MCF-7 cells (Table 8). The oxidovanadium(IV) salts with phen(H)^+^ and bpy(H)^+^ exhibited relatively higher cytotoxicity than the others. It is worth noting that the 1,10-phenanthrolinium analogue exhibited the highest activity, while the acridinium salt, which also comprises three conjugated aromatic rings but with a different ring arrangement, showed the lowest toxicity for all the cells investigated.

Previous reports have shown that the intercalating properties of nitrogen-containing heterocyclic compounds, which result in DNA damage, increase with the number of heterocyclic moieties. Therefore, VO^2+^ complexes with phen ligands are typically more effective in DNA cleavage than their bpy-containing counterparts [17,18,19,20]. This study demonstrates that the anticancer effect of the tested compounds is not only enhanced by the number of aromatic rings of the counterions [21,22] but also by their mutual geometric configuration. The IC_50_ parameters of the [VO(nta)(H_2_O)]^−^ complexes differ depending on the cations present. Specifically, complexes with cations containing three aromatic rings (phen, acr) exhibit different parameters compared to those with cations containing two aromatic rings (Q, bpy).

Unfortunately, at concentrations above 10 μM for (I), (II), and (IV), and above 1 μM for (III), the compounds have stronger effects on both normal (HaCaT) and tumoral (PC3 and MCF-7) cells. The selectivity indexes, calculated as the ratio of IC_50_ for the normal cell line and IC_50_ for the cancer cell line, are low and indicate a lack of selectivity for both types of cells (Table 8).

### 2.3. Cell Cycle Analysis

Although the investigated compounds do not appear to be potential antitumor agents, we examined the possible mechanism of their cytotoxic action by analyzing the cell cycle of breast (MCF-7) and prostate (PC3) cancer model cells (Supplementary Figures S1–S4).

Changes in the cell cycle distribution were not seen in the low, non-toxic concentration range of the complexes compared with the control. However, higher concentrations of compounds caused slight changes in the cell cycle phase distribution. In MCF-7 cells, compounds **I** and **II** inhibited cell growth by inducing a block in the G_2_/M phase of the cell cycle, whereas compounds **III** and **IV** arrested the cell cycle in the G_0_/G_1_ phase.

In PC3 cells, all compounds tested at higher concentrations caused a slight increase in the G_0_/G_1_ phase. At the same time, a decrease in the cell population in the G_2_/M phase was observed. Thus, in contrast to sodium metavanadate, a simple inorganic salt that induced G_2_/M cell cycle arrest in prostate PC3 cancer cells [23], the tested compounds are thought to have a cytotoxic effect resulting from G_0_/G_1_ cell cycle arrest. However, it is important to note that the impact of oxidovanadium(IV) complexes on cell progression depends on several factors, including the type of organic ligand [9]. Some oxidovanadium(IV) complexes with bpy and phen, namely [VO(ox)(bpy)(H_2_O)], [VO(ox)(phen)(H_2_O)], [VO(ida)(bpy)](H_2_O)_2_, (phen)[VO(ida)(phen)](H_2_O)_4_, and [phenH][VO(nta)(H_2_O)](H_2_O)_2_ (ox = oxalate ligand, bpy = 2,2′-bipyridine, phen = 1,10-phenanthroline, ida = iminodiacetate ligand, nta = nitrilotriacetate ligand), were reported to arrest the cell cycle in the S and G_2_/M phases in hepatocellular carcinoma cell lines [10]. Some other vanadium(IV) complexes with the phen ligand, [VO(satsc)(phen)] (satsc = salicylaldehyde thiosemicarbazone, phen = 1,10-phenanthroline) and [VO(3,5-dibrsatsc)(phen)] (3,5-dibrsatsc = 3,5-dibromosalicylaldehyde thiosemicarbazone), have been found to cause the G_0_/G_1_ cell cycle arrest in human hepatoma cell lines (BEL-7402, HUH-7 and HepG2) [24].

The examples above demonstrate that selecting the appropriate types of heterocyclic organic bases to act as auxiliary ligands (neutral complexes) or counterions (salt-type complexes) creates opportunities to design new coordination compounds with interesting physicochemical and biological properties.

## 3. Materials and Methods

### 3.1. Reagents

The reagents (Sigma-Aldrich, Poznań, Poland) used for the chemical studies were of analytical grade and were used without further purification. They were as follows: VO(acac)_2_ (≥98%), nitrilotriacetic acid (H_3_nta) (≥99%), acridine (≥97%), and quinoline (≥99%).

### 3.2. Synthesis of [QH][VO(nta)(H_2_O)](H_2_O)_2_ (***I***) and [(acr)H)][VO(nta)(H_2_O)](H_2_O)_2_ (***II***)

The synthesis of the complexes was carried out similarly to the methods used to obtain compounds with 2,2′-bipyridyl and 1,10-phenanthroline as the cation [2,3]. H_3_nta (0.01 mol; 1.91 g) and VO(acac)_2_ (0.01 mol; 2.66 g) were mixed and dissolved in 40 mL of water. After refluxing the mixture for approx. 0.5 h, the hot solution was filtered and cooled. The appropriate organic compound was then added (0.01 mol; 1.29 g quinoline, or 1.79 g acridine). To eliminate H_2_acac by evaporation, the mixtures were concentrated by heating. The solutions were allowed to crystallize at room temperature. Within 10 days, [QH][VO(nta)(H_2_O)](H_2_O)_2_ was obtained in the form of blue crystals, whereas the synthesis of [(acr)H)][VO(nta)(H_2_O)](H_2_O)_2_ gave light green fiber-like structures. The composition of the compounds studied was determined by elemental analysis of carbon, hydrogen, and nitrogen (Vario EL analyser Cube CHNS).

Anal. Calcd for [QH][VO(nta)(H_2_O)](H_2_O)_2_ (**I**): C, 43.7%, H, 4.2%, N, 6.8%, Found: C, 43.5%, H, 4.2%, N, 6.7%.

Anal. Calcd for [(acr)H][VO(nta)(H_2_O)](H_2_O)_2_ (**II**): C, 46.6%, H, 4.5%, N, 5.7%, Found: C, 46.4%, H, 4.4%, N, 5.6%.

Anal. Calcd for [(phen)H][VO(nta)(H_2_O)](H_2_O)_0.5_ (**III**): C, 46.6%, H, 4.5%, N, 5.7%, Found: C, 46.4%, H, 4.4%, N, 5.6%.

Anal. Calcd for [(bpy)H][VO(nta)(H_2_O)](H_2_O) (**IV**): C, 42.9%, H, 4.3%, N, 9.4%, Found: C, 42.6%, H, 4.3%, N, 9.2%.

### 3.3. X-ray Measurements

Crystal data, data collection, and structural refinement details are summarized in Table 9. The crystal structure data were collected on an IPDS 2T dual beam diffractometer (STOE & Cie GmbH, Darmstadt, Germany) at 120.0(2) K with MoKα radiation of a microfocus X-ray source for 1 (GeniX 3D Mo High Flux, Xenocs, Sassenage, France), and CuKα radiation for 2. Crystals were cooled using a Cryostream 800 open-flow nitrogen cryostat (Oxford Cryosystems, Long Hanborough, UK). Data collection and image processing were performed with X-Area 1.75 [25]. Intensity data were scaled with LANA (part of X-Area) in order to minimize differences in intensities of symmetry-equivalent reflections. The structures were solved using the intrinsic phasing procedure implemented in SHELXT and all non-hydrogen atoms were refined with anisotropic displacement parameters by applying a full-matrix least-squares procedure based on F2 using the SHELX–2014 program package [26,27]. The Olex2 version 1.2.10 [28] and WinGx version 2023.01 [29] program suites were used to prepare the final version of CIF files. Olex2 [28] was used to prepare the figures. Hydrogen atoms were refined using the isotropic model with Uiso(H) values fixed to be 1.2 or 1.5 times Ueq of the atoms to which they were attached. In the crystal structure of (**II**), one of the acridinium cations was modeled as disordered over two positions. Three water molecules in the asymmetric unit of (**I**) were found to be highly disordered, with atoms in special positions, and could therefore not be satisfactorily modeled. They were removed from the electron density map using the OLEX solvent mask command.

### 3.4. IR Spectra

The IR spectra were recorded on the BRUKER IFS 66 spectrophotometer in a KBr pellet over the 4400–650 cm^−1^ range (Supplementary Figures S5–S7).

### 3.5. Biological Studies

#### 3.5.1. MTT Assay

The MTT assay was used to determine the cytotoxicity of the compounds tested against MCF-7 (breast cancer cells), PC3 (prostate cancer cells), and HaCaT (human keratinocytes) lines. Cells were seeded in 96-well plates at a density of 4000 per well and incubated overnight (37 °C, 5% CO_2_). The cells were then treated with the compounds in the concentration range of 0.1 to 300 µM (aqueous solutions). The wells to which the vehicle alone (H_2_O) was added served as controls. The prepared plates were further incubated for 48 h. Then, aqueous MTT salt solutions (4 mg/mL, 25 µL/well) were added and, after 3 h incubation, the formazan product was dissolved in DMSO. Absorbance was measured at 570 nm, and the wavelength of 660 nm was used as a reference (EnSpire microplate reader, Perkin Elmer Lambda 650 (Waltham, MA, USA). The cell viability of the control was assumed to be 100%. Three independent experiments were performed in three replicates. The results were statistically analyzed using GraphPad Prism 7 (one-way ANOVA, Dunnett’s multiple comparison test).

#### 3.5.2. LDH Assay

The cells were incubated with the tested compounds in aqueous solutions at a concentration range of 0.1–200 µM and incubated at 37 °C with 5% CO_2_ for 48 h. After the appropriate incubation time, the level of LDH in the supernatant was analyzed using the CyQUANT LDH Cytotoxicity Assay from Thermo Fisher, following the manufacturer’s protocol. The solutions were then analyzed using a 96-well plate reader at 490 nm. LDH release was expressed as a percentage of the maximum release resulting from cell lysis (assumed to be 100%). Three independent experiments were carried out with three repetitions each. The results were statistically analyzed using GraphPad Prism 7 (one-way ANOVA, Dunnett’s multiple comparisons test).

#### 3.5.3. Cell Cycle Analysis

The cells were treated with the studied compounds in aqueous solutions at concentrations of 10, 50, and 100 µM. After incubation at 37 °C with 5% CO_2_ for 24 h, the cells were dissociated using Accutase solution, fixed with ice-cold 70% ethanol, and stained with propidine iodate for 30 min using Guava Cell Cycle Reagent from Luminex. The cells were then analyzed using flow cytometry with a Guava easyCyte 12 from Merck. The scatter plot FSC (Forward Scatter) versus the DNA content of the cell sample was used to gate the cells and exclude cell debris and cell aggregates. The GuavaSoft 3.3 software (InCyte mode) was used to evaluate the cells in different phases of the cell cycle.

## 4. Conclusions

Two new nitrilotriacetate oxidovanadium(IV) complexes with quinolinium and acridinium cations were successfully synthesized and their crystal structures were described based on X-ray measurements. Furthermore, the general structural features of complexes with some N-heterocyclic aromatic cations were compared and discussed. Biological studies have proven that newly synthesized complexes exhibit anticancer effects in a dose-dependent manner on prostate cancer (PC3) and breast cancer (MCF-7) cell lines. Here, higher concentrations of compounds led to changes in the cell cycle distribution, with different effects in MCF-7 and PC3 cells. In MCF-7 cells, compounds I and II blocked the G_2_/M phase, while compounds III and IV arrested the cell cycle in the G_0_/G_1_ phase. In PC3 cells, all compounds at higher concentrations increased the G_0_/G_1_ phase. Our studies have shown that the number of conjugated aromatic rings in the counterion is not the only factor influencing the anticancer activity of the tested salts. The arrangement of these rings also plays a key role in affecting the biological action. Unfortunately, our investigation found that the studied complexes lack selectivity, meaning they do not distinguish between cancer cells and normal human keratinocytes (the HaCaT cell line), which is a critical issue in developing effective anticancer treatments. In conclusion, our studies on vanadium compounds have brought some understanding of their physicochemical properties and biological activities, but unfortunately they indicate that the compounds presented in this paper cannot stand as effective anticancer agents for the treatment of prostate and breast cancer cells in vitro.

## Figures and Tables

**Table 1:** Anticancer properties of some nitrilotriacetate oxidovandium(IV) salts.

Structure	Antitumor Activities	References
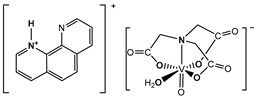	Cell lines: Human HepG2 and SMMC-7721 hepatocellular carcinoma cell lines Activity: Inhibition of cell proliferation. The IC_50_ values (the concentration for a 50% growth inhibition) are 42.46 μM for SMMC-7721 cells and 101.62 μM for HepG2 cells A relatively high cytotoxic effect (36.11 μM for SMMC-7721 and 107.79 μM for HepG2) was reported for the free phen ligand. The nta and bpy ligands did not reveal significant cytotoxicity (IC_50_ > 400 μM for HepG2 and SMMC-7721 cell lines)	[10]
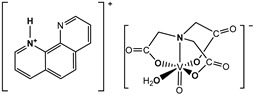 and 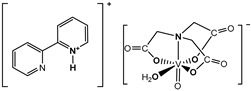 and 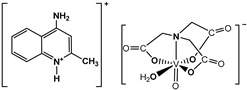	Cell lines: Human pancreatic ductal adenocarcinoma cell line (PANC-1), non-tumor human immortalized pancreas duct epithelial cells (hTERT-HPNE) Activity: Selective cytotoxicity of the complexes was observed for PANC-1 cells but their action was slightly lower than gemcitabine (a positive control, commonly used in pancreatic cancer treatment) Inhibition of autophagy process in selective cytotoxic concentration. The cell cycle arrest in the G_2_/M phase is associated with mitotic catastrophe. Induction of a mixed type of cell death in PANC-1 cells, including apoptotic and necroptotic processes	[5]
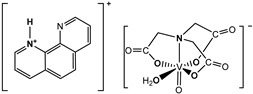 and 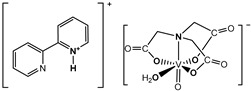	Cell lines: Human MG-63 and HOS osteosarcoma cell lines and the untransformed (normal) human osteoblast cell line (hFOB1.10) Activity: The compounds showed selectivity for malignant cells. The phenH salt exhibited a higher anti-proliferative activity towards MG-63 and HOS than the bpyH salt and cis-Pt(NH_3_)_2_Cl_2_ (a positive control)	[3]
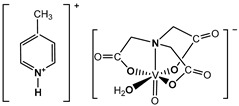	Cell lines: Human MG-63 and HOS osteosarcoma cell lines and the untransformed (normal) human osteoblast cell line (hFOB1.10) Activity: The cytotoxic effect was observed only at the higher concentration (>10 μM). Very low antiproliferative activity (MG-63 and HOS cells). Lack of selectivity for normal and malignant cells	[4]

**Table 2:** Selected structural data of [QH][VO(nta)(H_2_O)](H_2_O)_2_ (**I**).

Distance/Angle	Å, °	Distance/Angle	Å, °
V1A—O7A	1.611(3)	V1B—O7B	1.600(4)
V1A—O8A	2.001(3)	V1B—O8B	2.006(4)
V1A—O1A	2.001(3)	V1B—O1B	2.015(3)
V1A—O3A	1.984(3)	V1B—O3B	1.990(3)
V1A—O5A	2.002(3)	V1B—O5B	1.998(3)
V1A—N1A	2.332(3)	V1B—N1B	2.321(3)
O1A—V1A—O3A	90.57(1)	O1B—V1B—O3B	91.13(1)
O1A—V1A—O5A	150.27(1)	O1B—V1B—O5B	150.13(1)
O1A—V1A—O7A	101.91(1)	O1B—V1B—O7B	106.03(2)
O1A—V1A—O8A	85.02(1)	O1B—V1B—O8B	84.94(1)
O3A—V1A—O5A	87.37(1)	O3B—V1B—O5B	89.65(1)
O3A—V1A—O7A	96.19(2)	O3B—V1B—O7B	92.97(2)
O3A—V1A—O8A	161.96(1)	O3B—V1B—O8B	165.83(1)
O5A—V1A—O7A	107.80(1)	O5B—V1B—O7B	103.71(2)
O5A—V1A—O8A	87.85(1)	O5B—V1B—O8B	87.08(1)
O7A—V1A—O8A	101.84(1)	O7B—V1B—O8B	101.20(2)

**Table 3:** Selected structural data of [(acr)H][VO(nta)(H_2_O)](H_2_O)_2_ (**II**).

Distance	Å	Angle	°
V1—O8	1.594(5)	O1—V1—O3	92.25(2)
V1—O7	2.038(5)	O1—V1—O5	150.55(2)
V1—O1	2.020(5)	O1—V1—O7	86.90(2)
V1—O3	2.016(5)	O1—V1—O8	103.57(2)
V1—O5	2.009(4)	O3—V1—O5	87.27(2)
V1—N1	2.334(6)	O3—V1—O7	163.03(2)
		O3—V1—O8	95.20(2)
		O5—V1—O7	85.23(2)
		O5—V1—O8	105.79(2)
		O7—V1—O8	101.48(2)

**Table 4:** Hydrogen bonding interactions for [Q(H)][VO(nta)(H_2_O)](H_2_O)_2_.

*D*—H···*A*	*d*(*D*—H)	*d*(H···*A*)	*d*(*D*···*A*)	*<*(*D*—H···*A*)
O8B—H8BA···O2A	0.92	1.78	2.636 (5)	152.5
O8B—H8BB···O2B *^i^*	0.92	1.68	2.583 (5)	164.2
O8A—H8AA···O10C	0.92	1.74	2.624 (6)	158.9
O8A—H8AB···O3B *^i^*	0.92	2.36	2.928 (6)	119.5
N11B—H11B···O5B	0.96 (8)	1.71 (8)	2.666 (6)	172 (7)
N11A—H11C···O1A	0.88	2.20	2.862 (6)	131.8
N11A—H11C···O2A	0.88	2.26	3.083 (6)	154.6

Symmetry code: (*i*) *y*, −*x* + 1, −*z* + 1.

**Table 5:** Hydrogen bonding interactions for [acr(H)][VO(nta)(H_2_O)](H_2_O)_2_.

*D*—H···*A*	*d*(*D*—H)	*d*(H···*A*)	*d*(*D*···*A*)	*<*(*D*—H···*A*)
O8—H8A···O6 *^ii^*	0.84 (3)	1.80 (4)	2.597 (6)	157 (9)
O8—H8B···O2 *^iii^*	0.85 (3)	1.77 (3)	2.614 (6)	171 (9)
O10—H10B···O11	0.85 (3)	2.05 (10)	2.777 (10)	143 (14)
O10—H10C···O4	0.85 (3)	2.00 (9)	2.762 (8)	149 (15)
O11—H11A···O4 *^iv^*	0.85 (3)	2.13 (11)	2.839 (9)	141 (16)
O11—H11B···O10 *^v^*	0.85 (3)	1.90 (5)	2.735 (13)	166 (18)
N10—H10···O10	0.88	2.16	3.038 (15)	174.9
C22—H22···O3	0.95	2.32	3.234 (14)	162.6
N10A—H10A···O11	0.88	2.19	3.069 (14)	174.3
C22A—H22A···O7	0.95	2.39	3.297 (8)	160.1

Symmetry codes: (*ii*) −*x* + 1, *y* − 1/2, −*z* + 3/2; (*iii*) −*x* + 1, *y* + 1/2, −*z* + 3/2; (*iv*) *x* + 1, *y*, *z*; (*v*) *x* + 1/2, −*y* + 3/2, −*z* + 1.

**Table 6:** Stacking interactions in (**I**) [Å, °].

R(I)‧‧‧R(J)	Cg‧‧‧Cg	*α*	*β*	*d_p_*
Cg1‧‧‧Cg3 *^vi^*	3.691(3)	177.63(19)	177.3(6)	−3.432(5)
Cg2‧‧‧Cg4 *^vi^*	3.835(4)	177.3(2)	179.1(5)	−3.404(6)
Cg1‧‧‧Cg3	3.634(3)	2.37(19)	6.3(6)	3.415(5)
Cg2‧‧‧Cg4	3.676(4)	2.7(2)	7.7(6)	3.446(5)
Cg3‧‧‧Cg1 *^vii^*	3.691(3)	177.63(19)	177.3(6)	3.481(4)
Cg4‧‧‧Cg2 *^vii^*	3.835(4)	177.3(2)	179.1(5)	3.483(5)

Cg1, Cg2, Cg3, and Cg4 indicate the centroids of six-membered aromatic rings (R) depicted in Figure 4, *α* is a dihedral angle between planes I and J, *β* is an angle between the Cg(I) and Cg(J) vectors that is normal to plane I, and *d_p_* is a perpendicular distance of Cg(I) on the ring J plane. Symmetry transformations: (*vi*): *x*, *y*, − 1 + z; (*vii*): *x*, *y*, 1 + *z*.

**Table 7:** Stacking interactions in (**II**) [Å, °].

R(I)‧‧‧R(J)	Cg‧‧‧Cg	*α*	*β*	*d_p_*
Cg1‧‧‧Cg3 *^viii^*	3.740(9)	1.4(5)	2.5(11)	−3.324(13)
Cg2‧‧‧Cg3 *^viii^*	3.833(8)	0.4(5)	0.6(11)	−3.352(14)
Cg2‧‧‧Cg2 *^viii^*	3.702(8)	0.1(5)	0.1(9)	−3.353(14)
Cg3‧‧‧Cg2 *^viii^*	3.158(7)	0.6(5)	0.8(9)	−3.372(16)
Cg3‧‧‧Cg1 *^viii^*	3.645(8)	0.3(5)	0.6(12)	−3.364(12)
Cg1‧‧‧Cg3 *^ix^*	3.652(9)	1.4(5)	2.9(11)	3.335(17)
Cg2‧‧‧Cg3 *^ix^*	3.544(8)	0.4(5)	0.9(12)	3.342(14)
Cg2‧‧‧Cg2 *^ix^*	3.673(8)	0.1(5)	0.3(12)	3.340(12)
Cg3‧‧‧Cg2 *^ix^*	3.577(7)	0.6(5)	1.5(12)	3.336(11)
Cg3‧‧‧Cg1 *^ix^*	3.725(8)	0.3(5)	0.2(10)	3.343(12)

Cg1, Cg2, and Cg3 indicate the centroids of six-membered aromatic rings (R) depicted in Figure 5, *α* is a dihedral angle between planes I and J, *β* is an angle between the Cg(I) and Cg(J) vectors that is normal to plane I, and *d_p_* is a perpendicular distance of Cg(I) on the ring J plane. Symmetry transformations: (*viii*): − ½+ *x*, ½ − *y*, 1 − *z*; (*ix*): ½ + *x*, ½ − *y*, 1 − *z*.

**Table 8:** IC_50_ values and selectivity indexes (SI) of studied compounds. The selectivity index represents the ratio of IC_50_ for the normal cell line and IC_50_ for the cancer cell line after 48 h of treatment.

Compound	IC_50_ [µM]			SI	
	MCF-7	PC3	HaCaT	MCF-7	PC3
I	117.03	44.16	52.11	0.46	1.18
II	116.50	61.90	76.08	0.65	1.23
III	56.16	18.06	6.37	0.11	0.35
IV	86.93	40.78	30.15	0.35	0.74

**Table 9:** Crystal and structure refinement data of [QH][VO(nta)(H_2_O)](H_2_O)_2_ (**I**) and [(acr)H][VO(nta)(H_2_O)](H_2_O)_2_ (**II**).

Compound	[QH][VO(nta)(H_2_O)](H_2_O)_2_	[(acr)H][VO(nta)(H_2_O)](H_2_O)_2_
Empirical formula	C_30_H_34_N_4_O_17_V_2_ [+solvent]	C_19_H_22_N_2_O_10_V
Formula weight	824.49	489.37
Crystal system, space group	$\mathrm{tetragonal},\;I\bar{4}$	orthorhombic, *P* 2_1_ 2_1_ 2_1_
Unit cell dimensions [Å]	*a* = 31.8596(17)	*a* = 6.9331(13)
	*b* = 31.8596(17)	*b* = 9.950(3)
	*c* = 7.1243(4)	*c* = 29.644(7)
*V* (Å^3^)	7231.4 (9)	2044.9 (8)
Z	8	4
Radiation type	Mo *Kα*	Cu *Kα*
µ (mm^−1^)	0.60	4.62
Crystal size (mm)	0.52 × 0.06 × 0.05	0.57 × 0.06 × 0.03
Absorption correction	−	Multi-scan STOE LANA, absorption correction by scaling of reflection intensities [30]. Afterwards, a spherical absorption correction was performed within STOE LANA
*T*_min_, *T*_max_		0.318, 0.818
No. of measured, independent and observed [I>2σ(*I*)] reflections	7652, 5666, 4355	17,394, 3485, 3351
*R*_int_	0.040	0.049
(sin θ/λ)_max_ (Å^−1^)	0.596	0.593
*R*[*F*^2^ > 2σ(*F*^2^)], *wR*(*F*^2^), *S*	0.043, 0.091, 0.96	0.058, 0.155, 1.09
No. of reflections	5666	3485
No. of parameters	488	328
H-atom treatment	H atoms treated by a mixture of independent and constrained refinement	H atoms treated by a mixture of independent and constrained refinement
Δρ_max_, Δρ_min_ (e Å^−3^)	0.33, −0.26	0.51, −0.69
Absolute structure	Refined as an inversion twin	Flack x determined using 1321 quotients [(I+) − (I−)]/[(I+) + (I−)] [31]
Absolute structural parameter	0.09 (3)	−0.006 (4)
CCDC number	2341557	2341558

## Data Availability

The data presented in this study are available on request from the corresponding author.

## Supplementary Materials

The following supporting information can be downloaded at: https://www.mdpi.com/article/10.3390/molecules29122924/s1, Figure S1: The cell cycle distribution of MCF-7 cells after 24 h treatment with tested compounds (I-IV). Results are shown as the mean ± standard deviation (SD) of three independent experiments performed in triplicate; Figure S2: The histograms of MCF-7 cell cycle distribution after 24 h incubation with tested compounds. Histogram regions correspond to the G_0_/G_1_, S and G_2_/M phases; Figure S3: The cell cycle distribution of PC3 cells after 24 h treatment with tested compounds (I-IV). Results are shown as the mean ± standard deviation (SD) of three independent experiments performed in triplicate; Figure S4: The histograms of PC3 cell cycle distribution after 24 h incubation with tested compounds (I-IV). Histogram regions correspond to the G_0_/G_1_, S and G_2_/M phases; Figure S5: The IR spectra of H_3_nta (1) and Na_3_nta (2); Figure S6: The IR spectrum of [QH][VO(nta)(H_2_O)](H_2_O)_2_ (I).; Figure S7: The IR spectrum of [(acr)H][VO(nta)(H_2_O)](H_2_O)_2_ (II).
