# Supplementary material for: Exploring the Antitumor Efficacy of N-Heterocyclic Nitrilotriacetate Oxidovanadium(IV) Salts on Prostate and Breast Cancer Cells
Source: Molecules. 2024 Jun 19;29(12):2924. doi: 10.3390/molecules29122924 (PMC11206760; doi:10.3390/molecules29122924)
Supplement: Supplementary file 1 [file molecules-29-02924-s001.zip › molecules-3029044-supplementary.pdf]

Supplementary Information

*Article*

# Exploring the Antitumor Efficacy of N-Heterocyclic Nitrilotriacetate Oxidovanadium(IV) Salts on Prostate and Breast Cancer Cells

Katarzyna Chmur [1], Aleksandra Tesmar [1], Magdalena Zdrowowicz [1], Damian Rosiak [2], Jarosław Chojnacki [2], Dariusz Wyrzykowski [1]

[1] Faculty of Chemistry; University of Gdańsk; Wita Stwosza 63; 80-308 Gdańsk; Poland;
[2] Department of Inorganic Chemistry, Gdansk University of Technology, Narutowicza 11/12, Gdańsk 80-233, Poland;
katarzyna.chmur@ug.edu.pl (K.C.); aleksandra.tesmar@ug.edu.pl (A.T.); magdalena.zdrowowicz@ug.edu.pl (M.Z.); damianrosiak91@gmail.com (D.R.); jaroslaw.chojnacki@pg.edu.pl (J.C.); dariusz.wyrzykowski@ug.edu.pl (D.W.)
* Correspondence: dariusz.wyrzykowski@ug.edu.pl

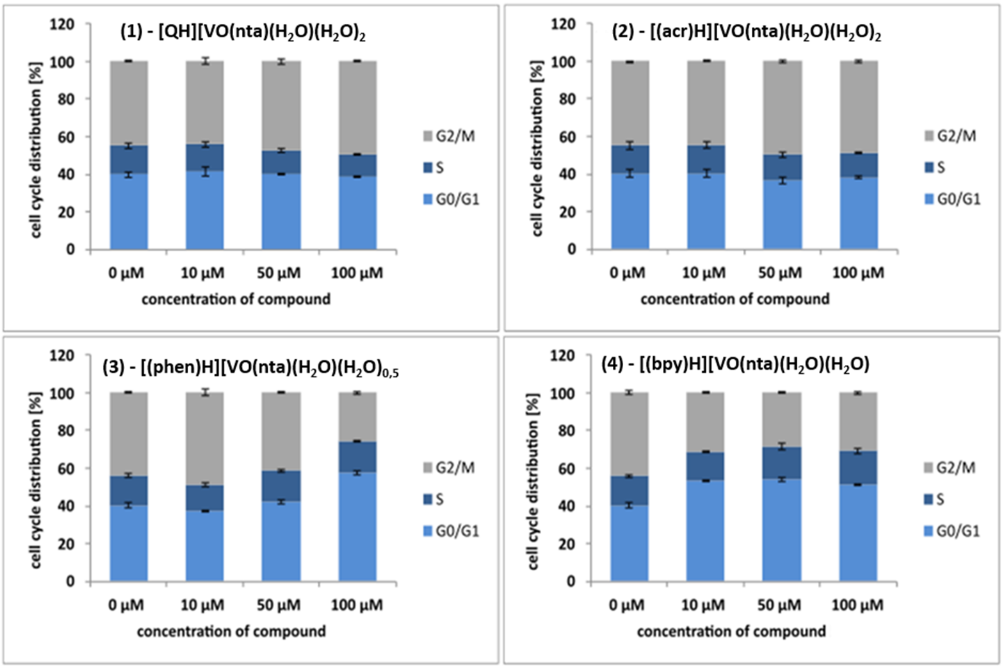


**Figure S1**. The cell cycle distribution of MCF-7 cells after 24 h treatment with tested compounds (I-IV). Results are shown as the mean ± standard deviation (SD) of three independent experiments performed in triplicate.

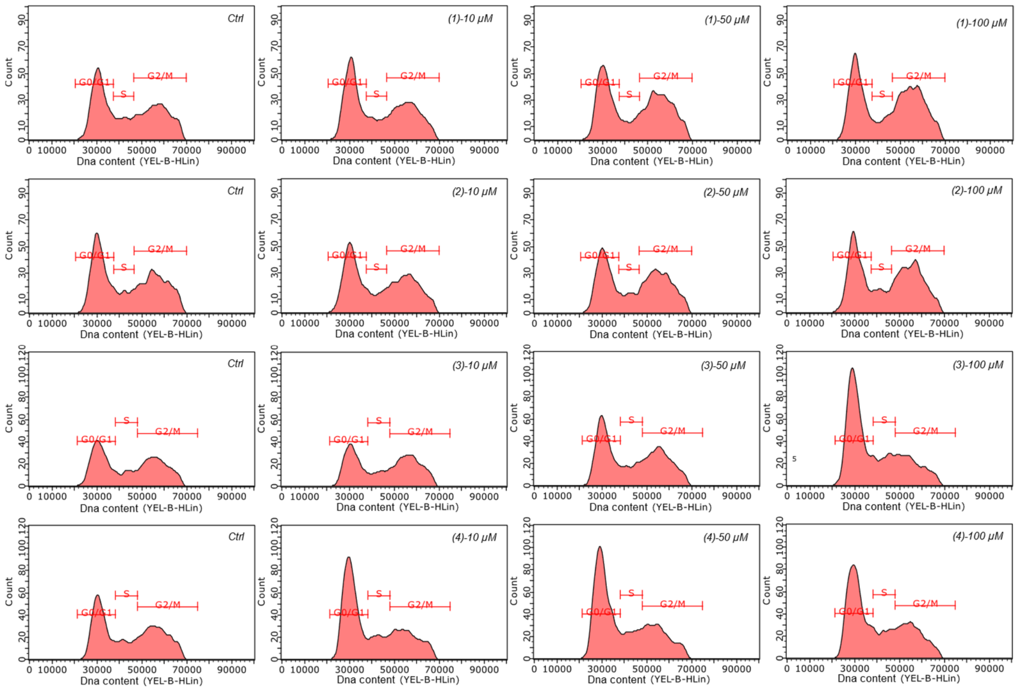


**Figure S2**. The histograms of MCF-7 cell cycle distribution after 24 h incubation with tested compounds. Histogram regions correspond to the $G_0/G_1$, S and $G_2/M$ phases.

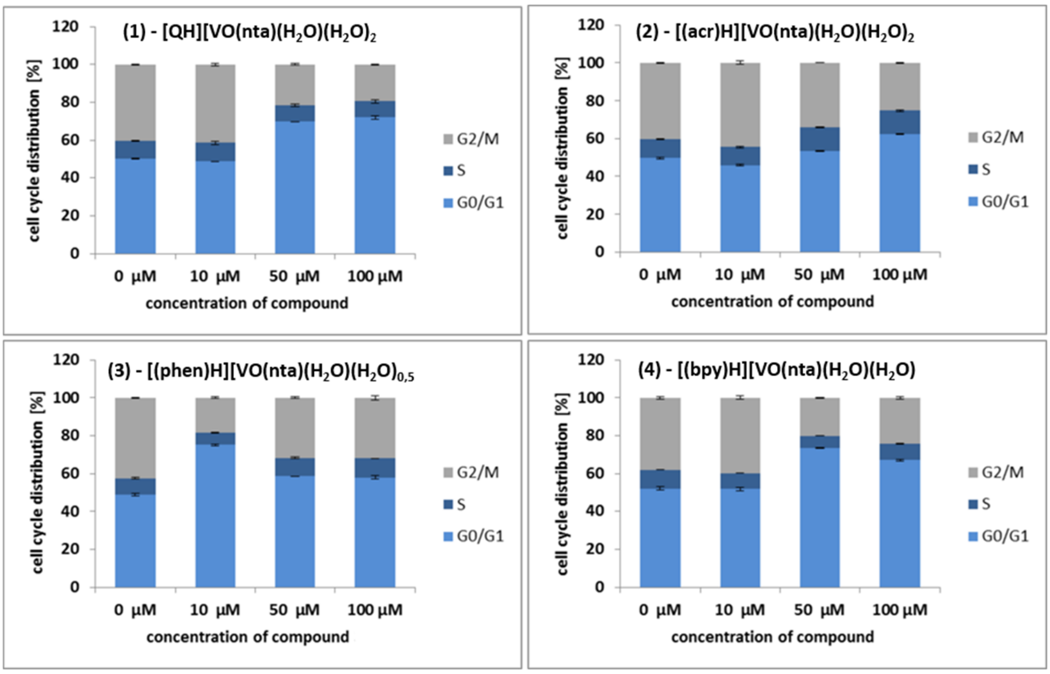


**Figure S3**. The cell cycle distribution of PC3 cells after 24 h treatment with tested compounds (I-IV). Results are shown as the mean ± standard deviation (SD) of three independent experiments performed in triplicate.

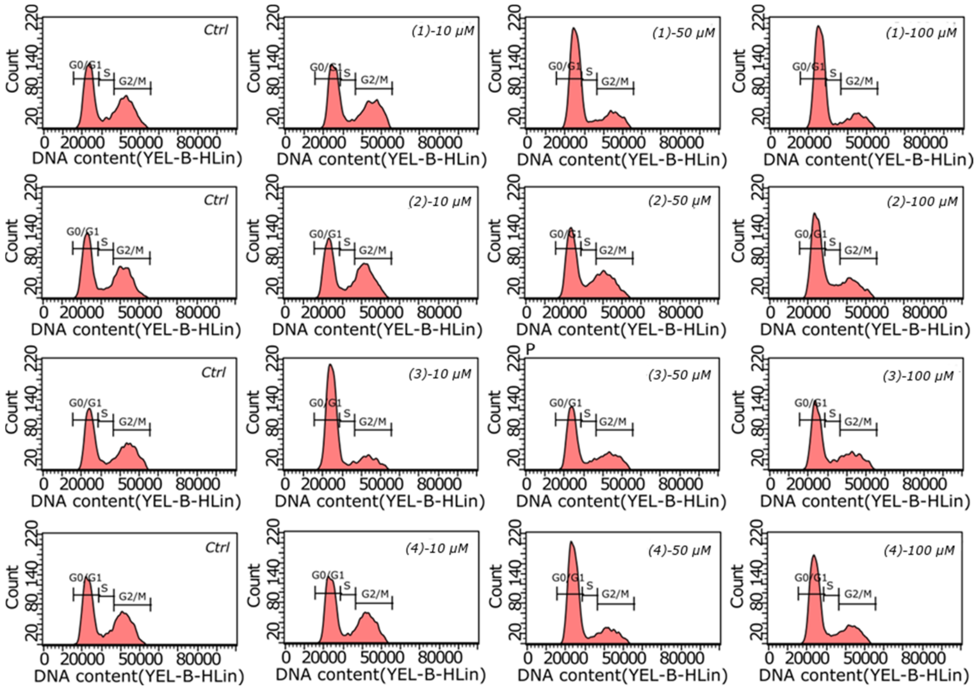


**Figure S4**. The histograms of PC3 cell cycle distribution after 24 h incubation with tested compounds (I-IV). Histogram regions correspond to the $G_0/G_1$, S and $G_2/M$ phases.

**IR spectra**

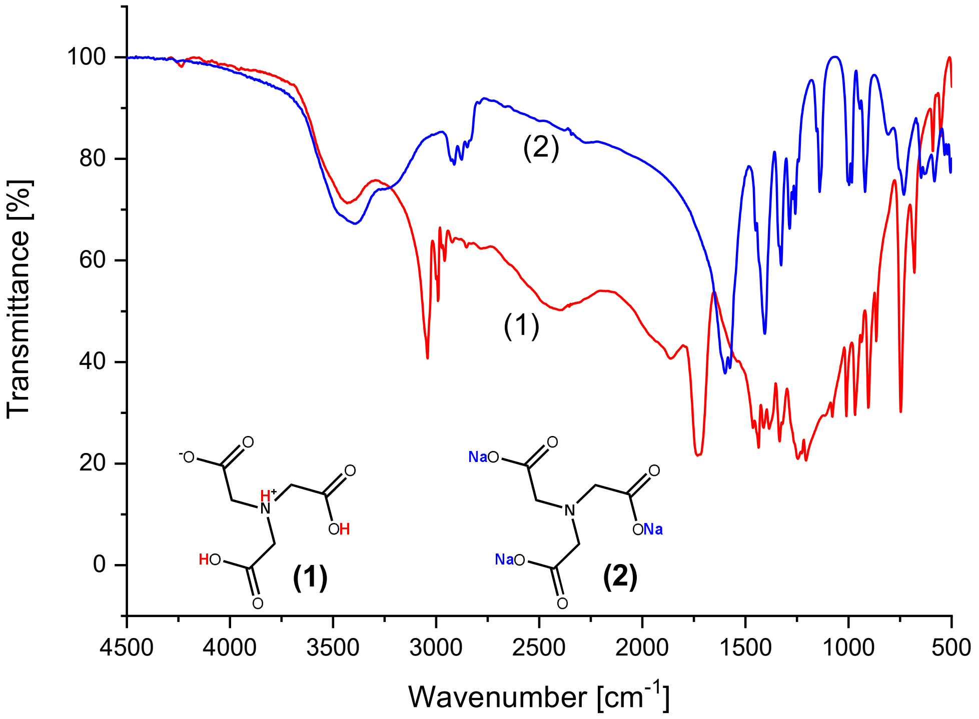


**Figure S5**. The IR spectra of $H_3$nta (**1**) and $Na_3$nta (**2**).

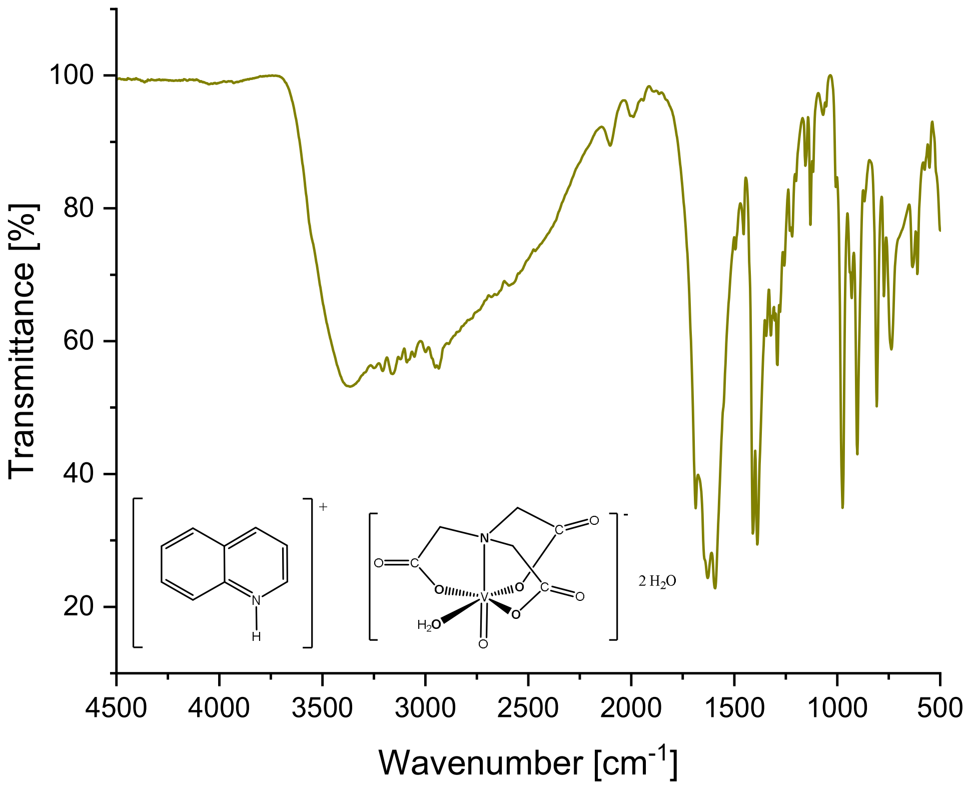


**Figure S6**. The IR spectrum of [QH][VO(nta)($H_2O$)]($H_2O$)$_2$ (**I**).

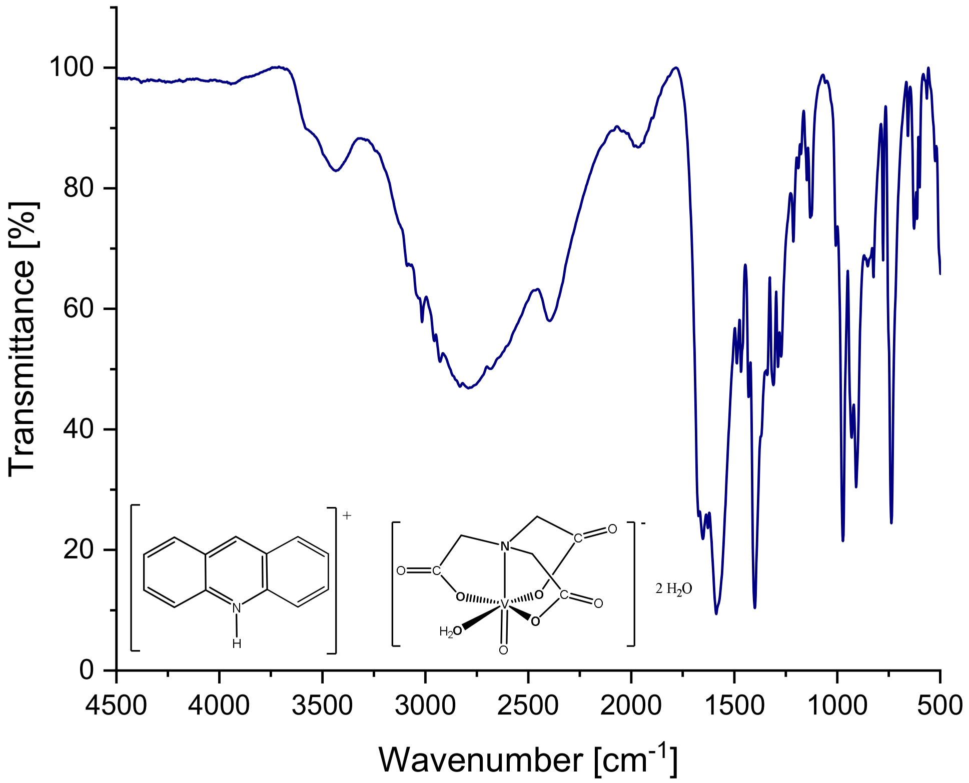


**Figure S7**. The IR spectrum of [(acr)H][VO(nta)($H_2O$)]($H_2O$)$_2$ (**II**).
